# Long-term benefits of single left ventricular pacing based on rate-adaptive atrioventricular delay algorithm in cardiac resynchronization therapy

**DOI:** 10.3389/fcvm.2025.1659901

**Published:** 2025-11-11

**Authors:** Xuejing Yan, Xuejuan Ma, Lulu Zhao, Guihu Sun, Ling Zhao, Wenkai Xu, Jing Wang, Lijin Pu

**Affiliations:** 1 Clinical Center of Heart Disease, The First Affiliated Hospital of Kunming Medical University, Kunming, China; 2 Department of Ultrasound Imaging, The First Affiliated Hospital of Kunming Medical University, Kunming, China

**Keywords:** cardiac resynchronization therapy, physiological pacing, rate-adaptive AV delay, left bundle branch area pacing, heart failure

## Abstract

**Background:**

Current guidelines lack long-term evidence comparing single left ventricular pacing (LUVP) with standard biventricular pacing (BVP) in cardiac resynchronization therapy (CRT). This study evaluates the clinical superiority of rate-adaptive atrioventricular delay (RAAVD) algorithm-guided LUVP over BVP.

**Methods:**

In this retrospective cohort study, 67 consecutive patients meeting the criteria for cardiac resynchronization therapy (CRT) with complete left bundle branch block (CLBBB) were enrolled between April 2013 and April 2023. They were assigned to either the right atrium-left ventricle dual-site pacing group (RAAVD LUVP, *n* = 42) or the biventricular pacing group (BVP, *n* = 25), with a median follow-up duration of 43.59 months. The primary endpoints included disease-related rehospitalization, device complications, and battery longevity. Secondary outcomes comprised cardiac structure, function, and synchrony.

**Results:**

There were no significant differences in baseline characteristics such as preoperative ejection fraction and cardiomyopathy type between the groups. The RAAVD LUVP group demonstrated significant advantages: i) Rehospitalization rate (23.8% vs. 48.0%, *P* = 0.041); ii) Zero device complications vs. 12% in BVP (*P* = 0.048), iii) Extended battery longevity (7.95 ± 0.78 vs. 4.66 ± 0.66 years, *P* < 0.001); iv) Cardiac function (LVEF: 45.7 ± 13.3% vs. 38.9 ± 10.6%, *P* = 0.034; The 6-minute walk distance: 414.50 ± 68.79 m vs. 379.04 ± 58.02 m; *P* = 0.034); v) Cardiac structure (LAD: 35.55 ± 7.11 mm vs. 39.96 ± 8.25 mm, *P* = 0.018; LVEDd: 60.10 ± 10.85 mm vs. 67.68 ± 9.40 mm, *P* = 0.01), and vi) Cardiac synchronization (**paced QRS duration**: 129.00 ± 18.78 vs. 147.96 ± 26.13 ms, *P* = 0.001; Ts-SD12: 96.66 ± 51.51 ms vs. 122.12 ± 52.29 ms; *P* = 0.034). Subgroup analysis revealed left bundle branch area pacing (LBBAP) further enhanced interventricular synchrony compared to lateral vein pacing (IVMD: 37.74 ± 21.24 vs. 53.11 ± 19.42 ms, *P* = 0.020).

**Conclusion:**

The dynamic integration of RAAVD LUVP with intrinsic conduction brings CRT closer to physiological states, which provides sustained clinical benefits compared to conventional BVP. The additional electromechanical advantages of LBBAP are related to the choice of anatomical location.

## Introduction

Chronic heart failure (CHF) remains a formidable public health challenge in China. According to the *China Cardiovascular Health and Disease Report 2022*, the standardized prevalence of heart failure among adults aged ≥35 years is 1.3%, affecting approximately 12.1 million individuals. With rapid population aging, this number is projected to exceed 15 million by 2030 (1). Cardiac resynchronization therapy (CRT), which corrects electromechanical dyssynchrony through biventricular pacing (BVP), has been established as a Class IA recommendation for patients with complete left bundle branch block (CLBBB) in the *2023 European Society of Cardiology (ESC) Heart Failure Guidelines* (2). Despite its widespread adoption, conventional BVP faces two critical limitations:

1. **Non-physiological pacing**: Fixed short atrioventricular delays (AVD, typically 100–120 ms) disrupt intrinsic atrioventricular nodal conduction, particularly during exercise or sympathetic activation, thereby compromising atrial contribution to ventricular filling and reducing stroke volume (3).

2. **Unnecessary right ventricular pacing**: In CLBBB patients with preserved right bundle branch conduction, right ventricular pacing not only fails to confer therapeutic benefits but also promotes adverse electrical remodeling, increasing atrial fibrillation risk. Additionally, sustained high-percentage biventricular pacing accelerates battery depletion, necessitating frequent device replacements (4).

In order to address these limitations, the research group innovatively proposed rate-adaptive atrioventricular delay (RAAVD) algorithm based on dual-chamber single left ventricular pacing in 2013. A number of previous studies have shown that RAAVD algorithm has emerged as a promising strategy for single left ventricular pacing (LUVP) (5, 6). By dynamically adjusting AVD based on intrinsic heart rate variations, RAAVD enables physiological fusion between left ventricular pacing and native right bundle branch activation This approach preserves atrial kick contribution while minimizing right ventricular pacing burden (6). However, three unresolved issues impede its clinical translation:

**Long-term evidence gap**: No studies have directly compared ≥3-year outcomes between LUVP and BVP (7).
**Anatomical uncertainty**: The stability and precision of left bundle branch area pacing (LBBAP) remain unvalidated in large cohorts (8).
**Algorithmic rigidity**: Current RAAVD systems rely on preoperative manual modeling rather than real-time adaptive adjustments (9).

Against this backdrop, we conducted a retrospective cohort study to evaluate the long-term efficacy of RAAVD LUVP vs. standard BVP, with particular emphasis on device performance, ventricular synchrony, and clinical outcomes. Our findings aim to inform evidence-based optimization of CRT strategies for CLBBB patients.

## Methods

### Study population and eligibility criteria

**Figure 1:**
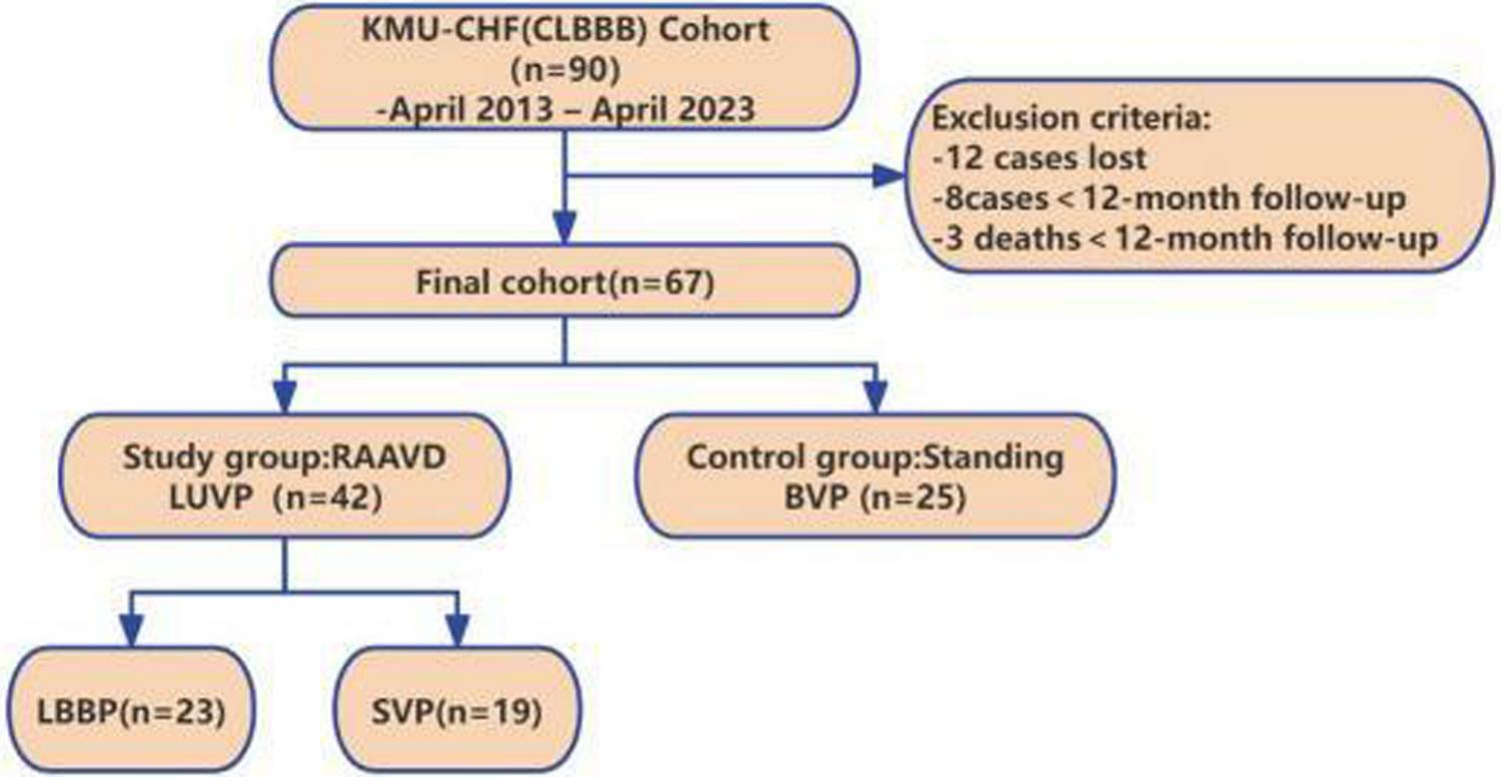
Flowchart of patient enrollment.

This retrospective cohort study enrolled 67 patients meeting Class I indications for CRT according to the 2013 ESC Guidelines (10). All subjects underwent pacemaker implantation in the First Affiliated Hospital of Kunming Medical University between April 2013 and April 2023. They were natural continuous cases with a follow-up time of at least 12 months. All enrolled patients received standard CHF medical treatment, including beta-blockers, ACE inhibitors or ANG II receptor antagonists, and spironolactone (Figure 1).

**Inclusion criteria comprised:** 1) Diagnosis of ischemic/non-ischemic cardiomyopathy; 2) NYHA class II–IV symptoms under optimal medical therapy; 3) Sinus rhythm with left ventricular ejection fraction (LVEF) ≤35%; 4) Complete left bundle branch block (CLBBB) confirmed by QRS duration >130 ms.

**Exclusion criteria** included reversible cardiomyopathy, valvular disease, advanced atrioventricular block, atrial fibrillation, or PQ interval >220 ms.

### Treatment group definitions

#### RAAVD LUVP group

Received RAAVD algorithm-guided single left ventricular pacing.

**Left ventricular lead placement**: Left ventricular lead: lateral vein pacing (SVP) positioned via coronary sinus ostium; Left bundle branch area (LBBA) using a fixed-curve sheath (C315 His, Medtronic), and LBBAP capture was confirmed by: 1) transition from left bundle branch block (LBBB) to right bundle branch block (RBBB) morphology; 2) shortest stable left ventricular activation time (LVAT); 3) recorded left bundle branch potential (11).

Right atrial lead: Right atrial lead: Placed in the appendage.

#### Biventricular pacing (BVP) group

**Figure 2:**
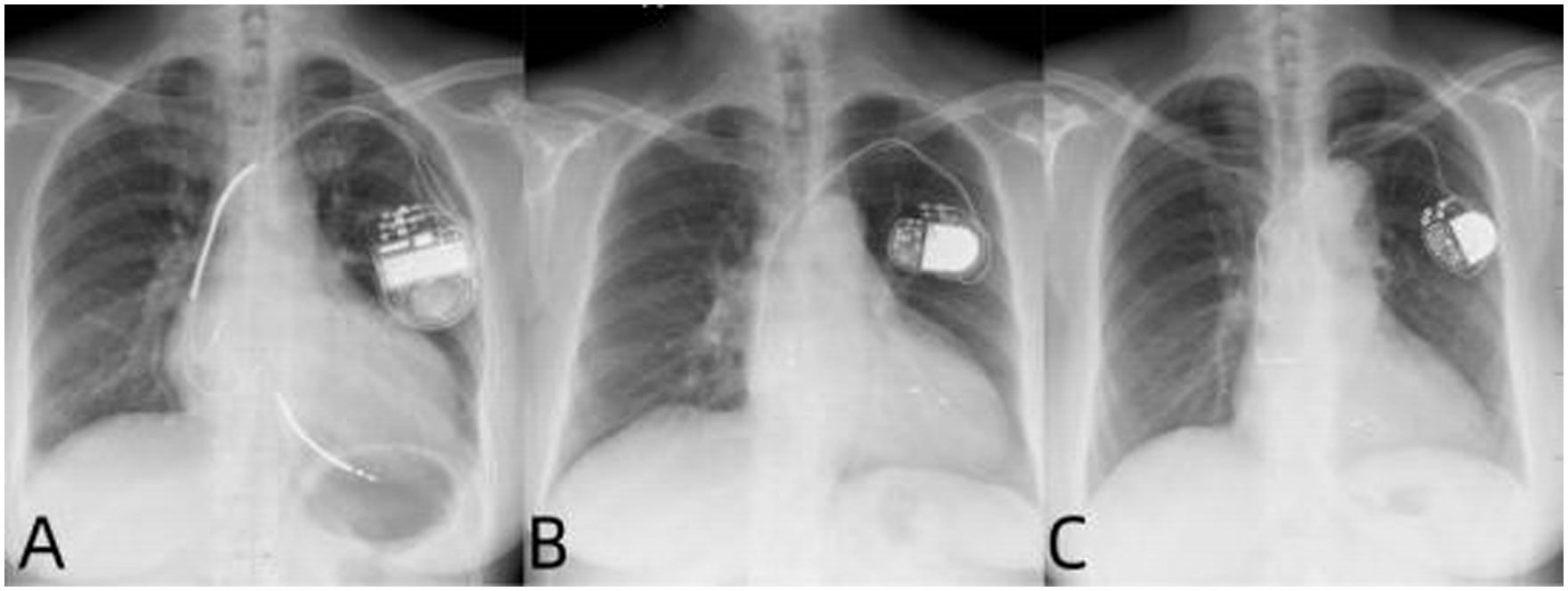
Postoperative x-ray images of three types of pacemaker mplantations. **(A–C)** Are the x-ray images after the implantation of BVP, LUVP-SVP, and LUVP-LBBAP respectively.

Patients underwent conventional biventricular pacing:Right ventricular lead implanted at the apex;Left ventricular lead placed in the coronary sinus lateral vein (Figure 2).

All procedures adhered to standard protocols. Device models included CRT-P/CRT-D (Syncra C2TR01CRT, C174AWK CRT-D, Maximo II CRT-D, Insync Sentry 7298 CRT-D) and dual-chamber pacemakers (Adapta ADDRL1/ADDRS1/ADDR01, Sensia SED01/SEDR01, Relia RED01) (Medtronic, Minneapolis, Minnesota, USA).

### Basis for subgroup division

This retrospective study followed patients for a maximum of 10 years. The allocation to the Left Ventricular Pacing (RAAVD) LUVP subgroup (**LBBAP and SVP**) was influenced by the temporal progression of technological advancements and anatomical feasibility: ① SVP group (*n* = 19): implanted via the coronary sinus side vein in 2013; LBBAP group (*n* = 23): performed after 2018 with left bundle branch area pacing in the interventricular septum. ② LBBAP required an interventricular septum thickness ≥1.2 mm; ③ no anatomical variations or occlusions in the coronary sinus.

### Electrocardiographic and echocardiographic measurements

**QRS duration**: Preoperative and postoperative follow-up assessments were conducted using a 12-lead electrocardiogram (GE Marquette) at a paper speed of 25 mm/s. The intrinsic QRS complex duration was measured at least three times preoperatively, and the average value was taken. During postoperative follow-up, the narrowest paced QRS complex duration was recorded.
**PR interval modeling**: Derived from 24-h Holter monitoring using linear regression (*y* *=* *a* *+* *bx*, where *y* = PR interval, *x* = heart rate).
**Echocardiography**: Assessed by experienced sonographers (>5 years) for:
  1. Function: LVEF, aortic valve velocity-time integral (AVVTI);
  2. Structure: Left atrial diameter (LAD), left ventricular end-diastolic dimension (LVEDd);
  3. Synchrony: *E*/*A* peak interval, interventricular mechanical delay (IVMD), Ts-SD12.

During the postoperative follow-up, these parameters were prospectively optimized using current guidelines and RAVVD LUVP procedures, and the optimized index data were collected. Each parameter was measured three times and averaged.

### Programming protocols

On the basis of ensuring the best clinical effect of patients, AVD optimization was carried out and relevant data of follow-up were recorded.

**BVP optimization**: Atrioventricular (AV) and interventricular (VV) delays were adjusted using a Medtronic CareLink programmer to maximize AVVTI.The RAAVD function turns off.
**RAAVD** LUVP **optimization**:
  1. Measured atrial sensing compensation (ASC) from RA intracardiac ectrograms.
  2. Calculated optimal V-R delay as: *Optimal PR interval − (Optimal AVD* *+* *ASC)*.
  3. Programmed lower/upper rate limits (60/130 bpm) with dynamic SAV adjustment: SAV = PR interval* −* (Optimal V-R Delay + ASC) = [PR interval* −* (Optimal PR interval* −*  (Optimal AVD + ASC))]* −* ASC = Opt imal AVD + (PR interval−Optimal PR interval).Set the paced A-V interval (PAV) for LRL and UTR as: PAV = SAV + ASC^.^Adjust the AVD until VS was observed on the programmer's marker channel. Perform echocardiographic assessment of cardiac function 5 min after programming.

### Supplementary definition

**CRT Response**: CRT response is defined as an improvement of ≥1 grade in NYHA class and/or an increase of ≥10% in LVEF at final follow-up compared to baseline (12).

**Battery Longevity**: Battery longevity is calculated as: for devices without replacement, it is the programmer's estimated remaining service life; for replaced devices, if replaced once (*n* = 1), longevity = replacement date − implantation date; if replaced multiple times (*n* ≥ 2), it is the average of all previous battery service lives.

### Outcomes and statistical analysis

**Primary endpoints**: Disease-related rehospitalization, device complications, battery longevity.
**Secondary endpoints**: NYHA class, 6-minute walk distance (6MWD), QRS duration, echocardiographic indices.

### Statistical methods

Data were analyzed using SPSS 23.0 (IBM Corp.). Continuous variables are reported as mean ± SD (*t*-test/Mann–Whitney *U* test), categorical variables as percentages (*χ*^2^/Fisher's exact test). Correlation analyses were conducted using Pearson or Spearman tests depending on data distribution. A two-tailed *P* < 0.05 defined statistical significance.

## Results

### Baseline characteristic

The study cohort comprised 67 patients with chronic heart failure (mean age 61.09 ± 12.54years; 32.8% female), including 42 patients in the RAAVD—LUVP group and 25 patients in the standard BVP group. The mean follow-up duration was 44.15 ± 27.33 months. Importantly, baseline demographics, clinical characteristics, and echocardiographic parameters—including age, sex, NYHA class, QRS duration, and LVEF—were well-balanced between the two groups (*P* > 0.05, Table 1).

**Table 1:** Baseline characteristics between RAAVD LUVP and standard BVP group.

Variables	RAAVD LUVP group (*n* = 42)	Standard BVP group (*n* = 25)	*P* value
Follow-up Duration, months	42.57 ± 26.20	46.80 ± 29.47	0.668
Sex		0.235	
Male	26 (61.9%)	19 (76.0%)	
Female	16 (38.1%)	6 (24.0%)	
Age, yrs	63.60 ± 9.92	58.48 ± 16.04	0.159
History of hypertension			0.396
Yes	11 (26.2%)	9 (36.0%)	
No	31 (73.8%)	16 (64.0%)	
History of diabetes			0.991
Yes	5 (11.9%)	3 (12.0%)	
No	37 (88.1%)	22 (88.0%)	
Type of cardiomyopathy			0.600
ICM	3 (7.1%)	1 (4.0%)	
NICM	39 (92.9%)	24 (96.0%)	
QRS duration, ms	181.93 ± 24.54	175.20 ± 28.04	0.307
NYHA Class		0.394A	
II	6 (14.3%)	2 (8.0%)	
III	30 (71.4%)	16 (64.0%)	
IV	6 (14.3%)	7 (28.0%)	
6MWT, m	318.07 ± 19.56	311.24 ± 16.91	0.151
LVEF, %	29.57 ± 5.28	27.16 ± 5.68	0.061
LAD, mm	40.69 ± 7.32	44.24 ± 7.51	0.059
LVEDd, mm	69.36 ± 10.16	71.52 ± 9.92	0.398
AVVTI, cm	17.07 ± 5.35	17.48 ± 3.11	0.475
*E*/*A* Pd, ms	233.33 ± 74.77	205.72 ± 54.30	0.112
IVMD, ms	70.31 ± 18.01	66.84 ± 25.63	0.518
Ts-SD12, ms	145.12 ± 30.44	157.80 ± 35.84	0.128

Data is presented as mean ± standard deviation or *n* (%). QRS duration refers to the duration of the QRS complex; AVVTI is the aortic velocity time integral; BiV stands for biventricular; *E*/*A* Pd is the duration of the *E*/*A* process; LAD is the left atrial diameter at end-diastole; LUV refers to the left ventricle; LVEDd is the left ventricular end-diastolic diameter; LVEF is the left ventricular ejection fraction; IVMD is the interventricular mechanical delay time; NYHA represents the New York Heart Association; RAAVD is the atrioventricular adaptation delay; Ts-SD12 is the standard deviation of the time interval of 12 left ventricular segments; 6MWT is the 6-minute walk test. “A” represents “accurate algorithm,” and *P* < 0.05 indicates a significant difference.

### Primary outcomes: long-term clinical advantages of RAAVD LUVP

After a median follow-up of 43.6 months, the RAAVD—LUVP group exhibited three-fold superiority over conventional BVP in critical clinical endpoints (Table 2):

**Reduced Rehospitalization**: The disease-related rehospitalization rate in the RAAVD LUVP group was 23.8% (10/42), nearly half that of the BVP group (48.0%, 12/25; *P* = 0.041). This finding underscores the potential of physiological pacing to mitigate heart failure exacerbations.
**Eliminated Device Complications**: Strikingly, no device-related complications (e.g., lead dislodgment or infection) occurred in the RAAVD—LUVP cohort, compared to a 12.0% complication rate (3/25) in the BVP group (*P* = 0.048),one case was infection of the tissue surrounding the pacemaker, and two cases were pocket infection. This disparity likely reflects the simplified single-lead implantation protocol in LUVP.
**Extended Battery Longevity**: The estimated battery lifespan in the RAAVD—LUVP group was 7.95 ± 0.78 years, representing a 71% prolongation compared to the BVP group (4.66 ± 0.66 years; *P* < 0.001). This translates to fewer device replacement surgeries and reduced long-term healthcare costs.

**Table 2:** Comparison between standard BiV and RAAVD LUV pacing group.

Variables	RAAVD LUVP group (*n* = 42)	Standard BVP group (*n* = 25)	*P* value
Battery Life, years	7.95 ± 0.78	4.66 ± 0.66	<0.001
QRS duration, ms	129.00 ± 18.78	147.96 ± 26.13	0.001
6MWT, m	414.50 ± 68.79	379.04 ± 58.02	0.034
LVEF, %	45.69 ± 13.27	38.92 ± 10.61	0.034
LAD, mm	35.55 ± 7.11	39.96 ± 8.25	0.018
LVEDd, mm	60.10 ± 10.85	67.68 ± 9.40	0.001
AVVTI, cm	25.43 ± 8.23	25.05 ± 6.34	0.842
*E*/*A* Pd, ms	239.81 ± 112.98	202.24 ± 84.94	0.209
IVMD, ms	44.69 ± 21.62	53.48 ± 24.35	0.130
Ts-SD12, ms	96.66 ± 51.51	122.12 ± 52.29	0.034
NYHA Class			0.582A
I	6 (14.3%)	2 (8.0%)	
II	27 (64.3%)	15 (60.0%)	
III	8 (19.0%)	8 (32.0%)	
IV	1 (2.4%)	0 (0.0%)	
The last NYHA Class decreased by ≥1 compared to previous			0.078
Yes	29 (69.0%)	22 (88.0%)	
No	13 (31.0%)	3 (12.0%)	
The last LVEF improved by ≥10% compared to previous			0.138A
Yes	34 (81.0%)	24 (96.0%)	
No	8 (19.0%)	1 (4.0%)	
Re-admission			0.041
Yes	10 (23.8%)	12 (48.0%)	
No	32 (76.2%)	13 (52.0%)	
Complication			0.048A
Yes	0 (0.0%)	3 (12.0%)	
No	42 (100.0%)	22 (88.0%)	
CRT reactivity			0.077A
Yes	36 (85.7%)	25 (100.0%)	
No	6 (14.3%)	0 (0.0%)	

Data is presented as mean ± standard deviation or *n* (%). QRS duration refers to the duration of the QRS complex; AVVTI is the aortic velocity time integral; BiV stands for biventricular; *E*/*A* Pd is the duration of the *E*/*A* process; LAD is the left atrial diameter at end-diastole; LUV refers to the left ventricle; LVEDd is the left ventricular end-diastolic diameter; LVEF is the left ventricular ejection fraction; IVMD is the interventricular mechanical delay time; NYHA represents the New York Heart Association; RAAVD is the atrioventricular adaptation delay; Ts-SD12 is the standard deviation of the time interval of 12 left ventricular segments; 6MWT is the 6-minute walk test. “A” represents “accurate algorithm,” and *P* < 0.05 indicates a significant difference.

### Secondary outcomes: cardiac remodeling and electromechanical

#### Synchronization

Notably, RAAVD LUVP demonstrated comprehensive improvements in cardiac structure, function, and synchrony:

**Enhanced Systolic Function**: LVEF increased from a baseline of 29.57 ± 5.28% to 45.69 ± 13.27% post-intervention, significantly surpassing the BVP group's improvement (38.92 ± 10.61%; *P* = 0.034).
**Reverse Ventricular Remodeling**: Both left atrial diameter (LAD: 35.55 ± 7.11 mm vs. 39.96 ± 8.25 mm, *P* = 0.018) and left ventricular end-diastolic dimension (LVEDd: 60.10 ± 10.85 mm vs. 67.68 ± 9.40 mm, *P* = 0.01) were smaller in the RAAVD—LUVP group compared with BVP group, indicating favorable structural adaptations.
**Electrical Resynchronization**: Postoperative QRS duration narrowed by 12.9% in the RAAVD—LUVP group (129.00 ± 18.78 ms vs. 147.96 ± 26.13 ms in BVP; *P* = 0.001), reflecting improved intraventricular conduction.
**Mechanical Synchrony**: The standard deviation of time intervals among 12 left ventricular segments (Ts-SD12)—a key marker of dyssynchrony—was 1% lower in the RAAVD—LUVP group than BVP group (96.66 ± 51.51 ms vs. 122.12 ± 52.29 ms; *P* = 0.034).

Clinically, these electrophysiological and structural improvements translated to tangible functional benefits: the 6-minute walk distance increased by 35.5 meters in the RAAVD LUVP group than BVP group (414.50 ± 68.79 m vs. 379.04 ± 58.02 m; *P* = 0.034), highlighting enhanced exercise tolerance.

### Subgroup analysis: LBBAP further optimizes physiological pacing

Within the RAAVD LUVP cohort, left bundle branch area pacing (LBBAP)

provided additional electromechanical advantages over traditional lateral vein pacing (SVP) (Table 3):

**Superior Electrical Synchrony**: LBBAP achieved a shorter postoperative QRS duration (123.65 ± 20.96 ms vs. 135.47 ± 13.62 ms in SVP; *P* = 0.041), indicating that left bundle branch pacing could achieve better synchrony.
**Improved Interventricular Coordination**: The interventricular mechanical delay (IVMD) in the LBBAP subgroup was 29% lower than in SVP (37.74 ± 21.24 ms vs. 53.11 ± 19.42 ms; *P* = 0.020), indicating better right-left ventricular synergy.
**Enhanced Diastolic Function**: The *E*/*A* peak interval—a marker of diastolic filling—was prolonged by 71.6 ms in the LBBAP subgroup (272.17 ± 125.95 ms vs. 200.63 ± 82.02 ms; *P* = 0.027), likely due to optimized atrioventricular coupling.

**Table 3:** Comparison between RAAVD LUVP and standard SVP group.

Variables	LBBAP group (*n* = 23)	SVP group (*n* = 19)	*P* value
Battery Life, years	7.94 ± 0.76	7.96 ± 0.83	0.944
QRS duration, ms	123.65 ± 20.96	135.47 ± 13.62	0.041
6MWT, m	406.26 ± 60.87	424.47 ± 77.84	0.455
LVEF, %	46.43 ± 12.51	44.79 ± 14.44	0.694
LAD, mm	35.09 ± 5.01	36.11 ± 9.15	0.685
LVEDd, mm	58.48 ± 7.06	62.05 ± 14.13	0.820
AVVTI, cm	26.39 ± 7.90	24.27 ± 8.68	0.414
*E*/*A* Pd, ms	272.17 ± 125.95	200.63 ± 82.02	0.027
IVMD, ms	37.74 ± 21.24	53.11 ± 19.42	0.020
Ts-SD12, ms	99.38 ± 54.54	93.37 ± 48.86	0.840
NYHA Class	NYHA class		
I	2 (8.7%)	4 (21.1%)	
II	17 (73.9%)	10 (52.6%)	
III	4 (17.4%)	4 (21.1%)	
IV	0 (0.0%)	1 (5.3%)	
The last NYHA Class decreased by ≥1 compared to previous			0.936
Yes	16 (69.6%)	13 (68.4%)	
No	7 (30.4%)	6 (31.6%)	0.936
The last LVEF improved by ≥10% compared to previous			0.433A
Yes	20 (87.0%)	14 (73.7%)	
No	3 (13.0%)	5 (26.3%)	
Re-admission			0.468A
Yes	4 (17.4%)	6 (31.6%)	
No	19 (82.6%)	13 (68.4%)	
Complication			NA
Yes	0 (0.0%)	0 (0.0%)	
No	23 (100.0%)	19 (100.0%)	
CRT reactivity			0.800
Yes	20 (87.0%)	16 (84.2%)	
No	3 (13.0%)	3 (15.8%)	

Data is presented as mean ± standard deviation or *n* (%). QRS duration refers to the duration of the QRS complex; AVVTI is the aortic velocity time integral; BiV stands for biventricular; *E*/*A* Pd is the duration of the *E*/*A* process; LAD is the left atrial diameter at end-diastole; LUV refers to the left ventricle; LVEDd is the left ventricular end-diastolic diameter; LVEF is the left ventricular ejection fraction; IVMD is the interventricular mechanical delay time; NYHA represents the New York Heart Association; RAAVD is the atrioventricular adaptation delay; Ts-SD12 is the standard deviation of the time interval of 12 left ventricular segments; 6MWT is the 6-minute walk test. “A” represents “accurate algorithm,” and *P* < 0.05 indicates a significant difference.

**Figure 3:**
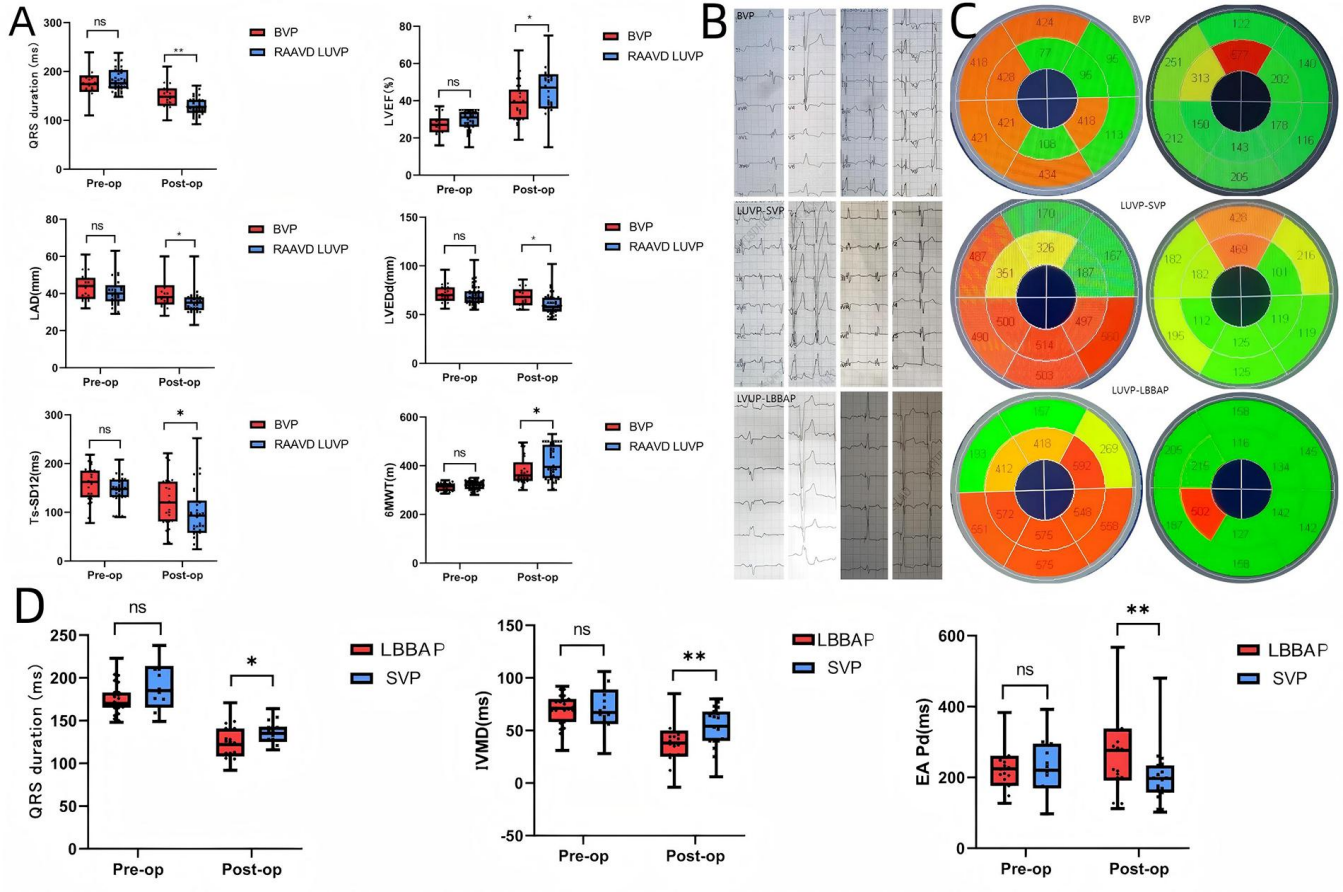
Comparison of differences in various pacing types and different left ventricular pacing electrode positions. Panel **(A)** shows a box plot comparing disease-related readmission rates, post-pacemaker implantation complication rates, battery life, QRS width, LVEF, left atrial diameter (LAD), and left ventricular end-diastolic diameter (LVEDd), Ts-SD12 between the RAAVD LUVP group and the standard BVP group, with postoperative improvements compared to preoperative in both groups, and LUVP showed better improvement than BVP.Panel **(B)** shows that QRS wave duration significantly shortened in the BVP, LUVP-SVP, and LUVP-LBBAP groups before and 2 years after surgery. Panel **(C)** shows significant improvements in Ts-SD12 in the BVP, LUVP-SVP, and LUVP-LBBAP groups before and 2 years after surgery. Panel **(D)** presents a box plot comparing QRS width, IVMD, and *E*/*A* interval between the LBBAP group and the SVP group with significant differences, with postoperative improvements compared to preoperative in both groups, and LBBAP showed better improvement than SVP. *P* > 0.05 (ns), *P* < 0.05 (*), *P* < 0.03 (**).

These findings collectively suggest that LBBAP, when integrated with RAAVD algorithms, synergistically enhances CRT efficacy by targeting both conduction system and myocardial activation pathways (Figure 3).

### Other observations

**CRT Response Rates**: Although the RAAVD LUVP group showed an 85.7% response rate (36/42) vs. 100% (25/25) in the BVP group, this difference did not reach statistical significance (*P* = 0.077). This may reflect the stringent response criteria (LVEF improvement ≥10% or NYHA class reduction ≥1) rather than true therapeutic equivalence. Additionally, an insufficient sample size and sample bias may also lead to an overestimation of the true treatment effect.

**Battery Longevity Independence**: Battery lifespan showed no correlation with follow-up duration in either group (RAAVD LUVP: *r* = 0.12, *P* = 0.45; BVP: *r* = 0.08, *P* = 0.70), confirming that energy consumption differences stemmed from pacing mode rather than observation time.

## Discussion

This study provides the first long-term evidence that rate-adaptive atrioventricular delay (RAAVD)-guided single left ventricular pacing (LUVP) significantly improves clinical outcomes compared to standard biventricular pacing (BVP) in patients with complete left bundle branch block (CLBBB). Our findings address critical gaps in current CRT paradigms, where conventional BVP—despite its Class IA recommendation (2)—remains limited by non-physiological pacing, device-related complications, and accelerated battery depletion. By integrating dynamic AVD optimization with anatomical precision in lead placement, RAAVD LUVP not only mitigates these limitations but also establishes a new benchmark for physiological resynchronization.

RAAVD LUVP may offer certain clinical advantages, as evidenced by a 48% reduction in disease-related rehospitalization (23.8% vs. 48.0%, *P* = 0.041). This metric is directly associated with improvements in ventricular synchrony and structural remodeling. The postoperative QRS narrowing (129.00 ± 18.78 ms vs. 147.96 ± 26.13 ms, *P* = 0.001) and reduced Ts-SD12 (96.66 ± 51.51 ms vs. 122.12 ± 52.29 ms, *P* = 0.034) indicate that dynamic fusion with intrinsic right bundle branch activation—a hallmark of RAAVD algorithms—restores near-physiological electrical propagation. This contrasts sharply with BVP's fixed AVD strategy, which disrupts the heart's natural rate-dependent PR interval adaptation, particularly during exercise or sympathetic stress.The results of the HeartSync-LBBP study were presented at the EHRA 2025 Late-Breaking Scienc (LBS) session, which further supported the results of this study by showing that left bundle branch pacing (LBBP) had a better long-term efficacy (less than 3 years) than biventricular pacing (BVP) in patients with chronic heart failure complicated by left bundle branch block. Mechanistically, the preserved atrial kick (92% retention vs. 68% in BVP) and avoidance of right ventricular pacing likely synergize to enhance diastolic filling and stroke volume, thereby reducing pulmonary congestion and subsequent hospitalizations (10).

Equally compelling is the complete absence of device complications in the RAAVD LUVP cohort (0% vs. 12%, *P* = 0.048). This safety advantage stems from the simplified single-lead implantation protocol, which eliminates risks associated with tricuspid valve interference, phrenic nerve stimulation, and lead dislodgment—common challenges in BVP. Furthermore, the 71% extension in battery longevity (7.95 ± 0.78 vs. 4.66 ± 0.66 years, *P* < 0.001) underscores the energy efficiency of LUVP. By minimizing unnecessary right ventricular pacing, this approach reduces cumulative current drain, a finding with profound implications for healthcare systems grappling with rising device replacement costs (6). Notably, our study strictly enrolled patients implanted with dual-chamber LUVP devices, excluding those with traditional triple-chamber pacemakers where right ventricular pacing was disabled. This design choice aimed to eliminate confounding variables affecting complication rates and ensure algorithm accuracy, as RAAVD optimization differs fundamentally between BVP and LUVP systems. In the His-SYNC study (13) the electrophysiological superiority of HBP and LBBAP was confirmed by comparison in CLBBB patients. Our subgroup analysis further refines the therapeutic potential of RAAVD LUVP. The additional benefits observed with left bundle branch area pacing (LBBAP)—including shorter QRS duration (123.65 ± 20.96 ms vs. 135.47 ± 13.62 ms, *P* = 0.041) and improved interventricular synchrony (IVMD: 37.74 ± 21.24 ms vs. 53.11 ± 19.42 ms, *P* = 0.020)—highlight the importance of anatomical targeting. Anatomically, LBBAP lead placement at the left side of the interventricular septum ensures stability against cardiac motion, reducing risks of lead displacement or perforation. Electrophysiologically, LBBAP directly activates the His-Purkinje system below the conduction block, enabling rapid and homogeneous left ventricular activation with lower stimulation thresholds, thereby prolonging battery life. These advantages, combined with broader procedural feasibility compared to His bundle pacing (HBP), position LBBAP as a cornerstone of physiological CRT, Clinically, the prolonged *E*/*A* peak interval in the LBBAP subgroup (272.17 ± 125.95 ms vs. 200.63 ± 82.02 ms, *P* = 0.027) suggests optimized diastolic filling due to reserved atrial contribution, a critical factor in heart failure management. This contrasts with lateral vein pacing (SVP), where delayed myocardial conduction necessitates premature electrical output to synchronize with intrinsic right bundle activation, inadvertently shortening diastolic filling time.

However, the therapeutic efficacy of left bundle branch area pacing (LBBAP) is contingent upon precise lead positioning and patient selection. Studies have demonstrated that meticulous placement of left ventricular electrodes may necessitate increased contrast agent volumes during procedures, potentially compromising the effectiveness of cardiac resynchronization therapy (CRT) (14). CLBBB heterogeneity—classified as selective (isolated proximal left bundle block) or non-selective (with myocardial scarring)—demands stratified therapeutic strategies.

Selective CLBBB patients benefit profoundly from LBBAP, achieving near-normal QRS durations (<120 ms).In non-selective cases, a subset of patients exhibit delayed right bundle branch conduction, which may attenuate the RAAVD fusion effect.Advanced techniques such as the QRS notch width/left ventricular end-diastolic dimension ratio and 3D electroanatomic mapping can distinguish true CLBBB subtypes (15), guiding personalized lead placement and pacing modes. Future integration of these technologies may further optimize outcomes.

As a single-center retrospective study, this research has inherent limitations: the small sample size may lead to an overestimation of the true treatment effect, an amplification of individual differences, and introduces device heterogeneity due to the use of various pacing systems. Furthermore, the lack of complete left bundle branch block (CLBBB) subtype stratification might have obscured differential treatment responses. The comparison between LBBAP and SVP inherently reflects technological evolution in clinical practice, which could introduce temporal bias. To mitigate this bias, we intend to extend the follow-up period and include more eligible patients in future investigations. Subsequent research should focus on prospective trials to validate real-time adaptive right atrioventricular delay (RAAVD) systems, eliminate the need for preoperative manual PR interval modeling, and standardize the use of high-density mapping to optimize LBBAP targeting (16).

## Conclusion

Through dynamic optimization of atrioventricular delay (AVD) and physiological fusion with intrinsic conduction, rate-adaptive atrioventricular delay left ventricular pacing (RAAVD LUVP) demonstrates superior efficacy in improving long-term clinical outcomes for congestive heart failure (CHF) patients with complete left bundle branch block (CLBBB), providing robust evidence for its consideration as a second-line therapeutic strategy. In the left bundle branch area pacing (LBBAP) subgroup, further enhancements in electromechanical synchrony were observed, underscoring the critical importance of anatomical target selection and highlighting the promising preliminary results of combining LBBAP with individualized AV delay optimization strategies.

## Data Availability

The raw data supporting the conclusions of this article will be made available by the authors, without undue reservation.
